# Diseases of the Pancreas

**Published:** 1905-06-24

**Authors:** 


### DISEASES OF THE PANCREAS.

**The Relation of the Pancreas to Malignant Disease.**—After a detailed account of the gross and minute anatomy of the pancreas, Hillier and Goodall[1] describe the changes it presents in relation to malignant disease under four headings:—(1) Primary carcinoma of the pancreas; (2) Secondary carcinoma of the pancreas; (3) Carcinoma invading the pancreas by extension from neighbouring organs, and (4) the structure of the pancreas in cases of malignant disease not directly affecting it. Primary carcinoma is present in three forms:—spheroidal celled arising from the acini; collumnar celled from the ducts, and squamous celled from the elements which compose the islands of Laugerhans. Carcinoma of the pancreas was observed secondary to malignant disease of the breast (four cases), of the stomach (two cases), of the rectum and uterus (one each). The secondary growth in each case seems to extend most rapidly along the lymph channels and the connective tissue. In secondary growths the islands of Langerhaus seem to be often more affected by the disease than other parts of the gland, whereas in primary growths the reverse is the case, except in the squamous celled carcinoma which begins in the islands of Laugerhans. It is suggested that the spaces seen in the midst of many of the islands are lymph channels, and it is these which convey the secondary malignant infection to the islands of Laugerhans. Carcinoma which invades the pancreas by direct extension commonly arises from one of two sources—the duodenum and the bile-ducts, and is in each case a columnar celled growth. In conditions of malignant disease not involving the pancreas, the latter nevertheless in about 40 per cent. of cases examined shows certain changes. These consist of fibrosis —a great thickening of the normally scanty connective tissue, and in the presence of some peculiar alveoli resembling fœtal pancreatic tissue. An analysis is added of 100 cases of primary carcinoma of the pancreas. From this it appears that 60 per cent. were males and 40 per cent. females. Of the cases in men, nearly a third occurred between the ages of 40 and 50, whereas in women no particular age was specially liable to the disease. In 73 per cent. the head of the gland was the seat of the primary disease, in about 65 per cent. wasting, jaundice and abdominal pain were the chief symptoms, in 4 per cent. glycosuria was present.

**Empirical Methods Adopted in the Treatment of Cancer.**—Myler[2] first reviews the question of oöphorectomy for inoperable cancer and shows that there is a fair prospect in women who have not arrived at the menopause, of inhibiting for a time the growth of secondary or recurrent deposits in the breast, but that the improvement in the cases he noted was only temporary, lasting for a few months. Cases of cancer of the uterus are not affected at all by removal of the ovaries. The use of thyroid extract and the application of high frequency currents gave no good results. The $x$-rays produced a marked improvement in superficial nodules and in ulcerated skin areas, but internal cancer was quite unaffected, and even in cases where the superficial nodules were disappearing death took place by internal dissemination. Three cases (one of the vulva and two of the breast) were treated by injections of Adamkiewicz cancroin. The injections caused great pain, and though persevered in for several months gave no sign of benefit. Violet leaves, marigold ointment, liquor ammoniæ fort, molasses, applied externally and Chian turpentine internally, have each and all failed to produce any effect on those cases in which they were tried at the Middlesex Hospital. As regards the general management of inoperable cases, it is noted how greatly patients benefit from the regular discipline and nursing which they get in a hospital, so that they seldom require large or frequent doses of morphine. Phenacetin and caffein are highly spoken of as an anodyne.

**Heredity in Cancer.**—Messrs. Hillier and Tritsch,[3] after reviewing the literature on the subject of heredity in malignant disease, proceed to give a table of the results obtained by inquiry among 3,000 patients in the cancer wards of the Middlesex Hospital. Of these 617 were men, in 50 of whom a family history of cancer was obtained, while 2,383 were women with 408 having family cancer. Karl Pearson[4] proceeds to analyse the statistics by a method of mathematical curves. He thus deduces (1) as to age incidence—that the liability to cancer in women begins about 16, reaches its climax at 46, and diminishes to 99. In men it begins at two, reaches its climax at 56, and diminishes to 91. (2) As to heredity. On comparing 3,000 non-cancerous patients with 3,000 cancerous, the proportion of those with cancerous relatives, although slightly greater in the latter case, the difference is practically negligible. Thus as far as these extensive statistics are concerned, the hereditary nature of cancer is not borne out. (3) As to the relation between tubercle and cancer, that cancer patients have no more hereditary history of tubercle than non-cancerous, but that a patient with cancer is rather more likely to contract tubercle than a non-cancerous.

**The Origin of "Potato" Tumours of the Neck from the Carotid Gland.**—Gilford[5] and Davis record three cases of malignant tumours of the neck all occurring in elderly men (52, 62, and 74). In two cases it was unilateral, in the third bilateral. The growth began beneath the upper third of the sterno-mastoid and rapidly infiltrated the neighbouring structures. One patient died after the removal of the growth with the common carotid and jugular vein, the second died from recurrence soon after removal, and the third was inoperable and had secondary growths in the liver. In all the cases the shape and consistence of the tumour resembled a raw potato; its microscopical structure consisted of alveoli of spindle endothelial cells in a loose connective tissue stroma. The carotid sheath was always penetrated by the growth at the point of bifurcation of the artery, and these fact leads to the conclusion that the growth is an endothelioma springing from the carotid "gland."

**Removal of Primary Hepatic Carcinoma.**—Freeman[6] describes a successful case of removal of carcinoma of the liver. Patient was a man, aged 27, who had had pain and dragging in the right hypochondrium for one year with occasional slight jaundice. A mass the size of a walnut was situated at about the locality of the gall-bladder. On opening the abdomen this was found to be part of a mass in the lower edge of the right lobe of the liver the size of the fist. Omental adhesions, but no secondary growths, were present. The growth was isolated from the liver by thrusting long narrow strips of gauze through the hepatic substance with long fine forceps. The free ends of each gauze loop were tied together so as to compress all the vessels in their grip. The growth was then cut out without hæmorrhage and the wound packed with gauze. The gauze ligatures were removed later with some difficulty and the patient discharged in six weeks and was well 16 months later. Microscopically the growth was an alveolar carcinoma.

[1] Archives of the Middlesex Hospital, Vol. II. [2] Ibid., p. 65. [3] Ibid., p. 104. [4] Ibid., p. 127. [5] Prac., Dec. 1904. [6] Amer. Jour. Med. Sci., Oct. 1904.

